# Changes in the Surface Expression of Intercellular Adhesion Molecule 3, the Induction of Apoptosis, and the Inhibition of Cell-Cycle Progression of Human Multidrug-Resistant Jurkat/A4 Cells Exposed to a Random Positioning Machine

**DOI:** 10.3390/ijms21030855

**Published:** 2020-01-28

**Authors:** Alisa Sokolovskaya, Ekaterina Korneeva, Danila Zaichenko, Edward Virus, Dmitry Kolesov, Aleksey Moskovtsev, Aslan Kubatiev

**Affiliations:** Department of Molecular and Cellular Pathophysiology, Institute of General Pathology and Pathophysiology, Baltiyskaya str. 8, 125315 Moscow, Russia; katya96korn@mail.ru (E.K.); danilamihailovich@mail.ru (D.Z.); virus.e.d.68@gmail.com (E.V.); maedros@bk.ru (D.K.); bioinf@mail.ru (A.M.); aslan.kubatiev@gmail.com (A.K.)

**Keywords:** microgravity, random positioning machine, cell cycle, apoptosis, Jurkat cells

## Abstract

Experiments from flight- and ground-based model systems suggest that unexpected alterations of the human lymphoblastoid cell line Jurkat, as well as effects on cell growth, metabolism, and apoptosis, can occur in altered gravity conditions. Using a desktop random positioning machine (RPM), we investigated the effects of simulated microgravity on Jurkat cells and their multidrug-resistant subline, Jurkat/A4 cells. The viability of Jurkat/A4 cells decreased after simulated microgravity in contrast with the Jurkat cells. At the same time, the viability between the experimental Jurkat cells and control Jurkat cells was not significantly different. Of note, Jurkat cells appeared as less susceptible to apoptosis than their multidrug-resistant clone Jurkat/A4 cells, whereas cell-cycle analysis showed that the percentage of Jurkat/A4 cells in the S-phase was increased after 72 and 96 h of RPM-simulated microgravity relative to their static counterparts. The differences in Jurkat cells at all phases between static and simulated microgravity were not significant. The surface expression of the intercellular adhesion molecule 3 (ICAM-3)—also known as cluster of differentiation (CD)50—protein was changed for Jurkat/A4 cells following exposure to the RPM. Changes in cell morphology were observed in the Jurkat/A4 cells after 96 h of RPM-simulated microgravity. Thus, we concluded that Jurkat/A4 cells are more sensitive to RPM-simulated microgravity as compared with the parental Jurkat cell line. We also suggest that intercellular adhesion molecule 3 may be an important adhesion molecule involved in the induction of leukocyte apoptosis. The Jurkat/A4 cells with an acquired multidrug resistance phenotype could be a useful model for studying the effects of simulated microgravity and testing anticancer drugs.

## 1. Introduction

Space experiments are of great value for the study of various physiological characteristics of the human body; health problems; and, in particular, cancer [1,2,3]. Microgravity allows one to explore the molecular and cellular mechanisms of the biological effects on cellular stress and the transformation of normal and tumor cells with a change in gravity [4,5,6].

Many reports have indicated that simulated microgravity affects cellular morphology, proliferation, apoptosis, invasion, and migration while inhibiting cancerous cell growth and invasion [6,7,8,9,10,11,12,13]. After simulated-microgravity exposure, apoptosis has been observed in glial cells [7], endothelial cells [8], thyroid cancer cells [9], and Jurkat cells [5]. Both real and simulated microgravity induces early alterations of the cytoskeleton in thyroid cancer cells [10], endothelial cells [11], glial cells [12], and human breast cancer cells MCF-7 [13]. In a suspension cell culture, conditions similar to microgravity can be established using different equipment such as a rotating wall bioreactor, a clinostat, a random positioning machine (RPM), and even magnets (magnetic levitation) [14].

The human leukemic T-cell line Jurkat is a stable cellular model that is easily able to be managed and is sensitive to real and simulated microgravity [15,16,17,18]. The changes in the expression and secretion of genes as well as proteins involved in cancer cell proliferation, metastasis, and survival shift the cells toward a less aggressive phenotype. For example, on chondrocyte cultures, microgravity led to an increase in the expressions of apoptotic proteins Fas/apoptosis antigen-1, p53, and Bax, and a decrease in the antiapoptotic protein Bcl-2 [4]. Interestingly, the increased expression of the Fas protein in the Jurkat human T-cell lymphoblastoid line was also noted during the space shuttle flights STS-80 and STS-95. It was concluded that changes in the cytoskeleton and lymphocyte metabolism, as well growth retardation during space flight, are associated with an increase in apoptosis and with the temporary overexpression of the apoptotic Fas/apoptosis antigen-1 protein [5]. 

Recently, in vitro microgravity has been of particular interest as a system for both studying cancer mechanisms and for testing anticancer drugs [6,19,20]. Multicellular spheroids are a model for studying the mechanisms of angiogenesis and pharmacological testing of chemotherapeutic agents [21,22,23]. 

Jurkat T-cells have a high proliferation rate, are moderately sensitive to most anticancer drugs and ionizing radiation, and do not express the p53 protein [24,25]. A multiresistant cell line, Jurkat/A4, which is a stable subline of Jurkat cells with an acquired multidrug resistance phenotype, constitutes a useful model for studying apoptotic resistance [26,27]. Jurkat/A4 cells retain the morphology of Jurkat, initially expressing low levels of FasR (cluster of differentiation (CD)95) and intercellular adhesion molecule 3 (ICAM-3) (CD50) on their cell surface. However, little is known about the effects of simulated microgravity on cells with a multidrug resistance phenotype.

To our knowledge, the effects of reduced gravity on the cellular responses and physiological effects of Jurkat/A4 have not yet been investigated. Therefore, in the present study, we investigated the effects of simulated microgravity on Jurkat cells and their multidrug-resistant subline, Jurkat/A4 cells.

## 2. Results

### 2.1. Effect of Simulated Microgravity on Cell Viability of Jurkat/A4 Cells

**Figure 1:**
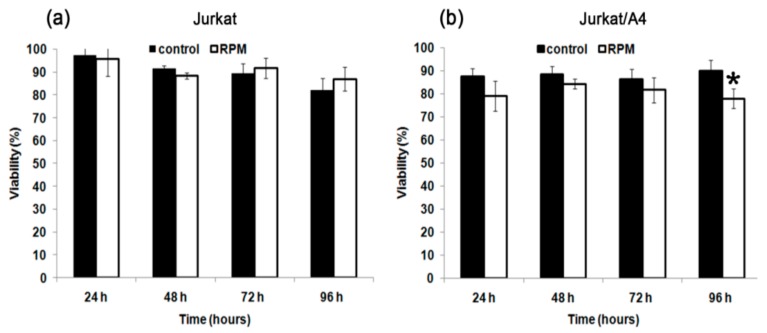
The effect of random positioning machine (RPM)-simulated microgravity on cell viability of Jurkat (**a**), and Jurkat/A4 cells (**b**). Cell viability was evaluated with a trypan blue exclusion assay. The results are expressed as means ± standard deviations. * *p* < 0.05, as compared with the static controls (*n* = 7).

The analysis of cell viability was performed using the vital dye trypan blue. The viability of Jurkat/A4 cells decreased after exposure to simulated microgravity in contrast with the Jurkat cells. After 96 h, the percentage of Jurkat/A4 alive cells had decreased under RPM-simulated microgravity conditions (76.0% ± 4.2%) relative to the static control cells (90.2% ± 4.2%) (*n* = 7; *p* < 0.05). At the same time, the viability profile between the experimental Jurkat cells and control Jurkat cells was not significant (Figure 1). 

### 2.2. Simulated Microgravity Induced Apoptosis of Jurkat/A4 Cells 

**Figure 2:**
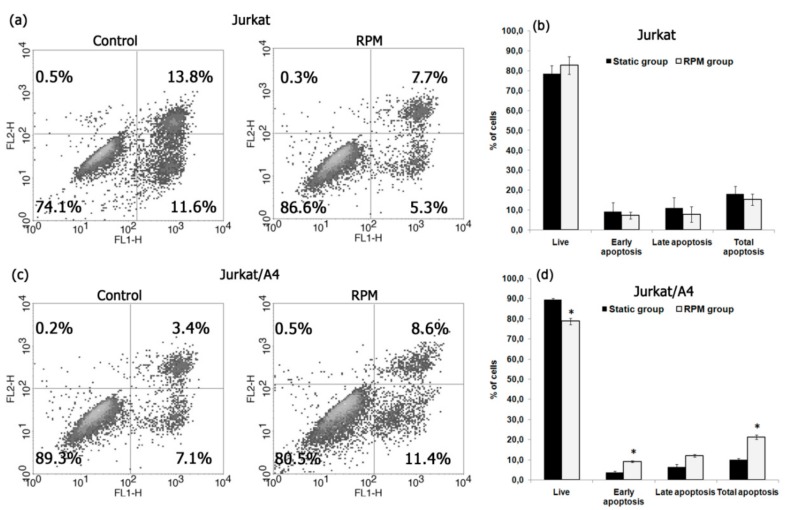
Apoptosis in Jurkat and Jurkat/A4 cells under simulated microgravity (96 h). Cells were stained with annexin V, conjugated, and evaluated for apoptosis as described in the Materials and Methods section. (**a**,**c**) Flow cytometric analysis of cells to assess apoptosis using annexin V labelling. Results are shown as percentages of viable cells (annexin V−/propidium-iodide (PI)−), early apoptotic cells (annexin V+/PI−), late apoptotic cells (annexin V+/PI+), and dead cells (annexin V−/PI+). The apoptosis rates were statistically evaluated. (**b**,**d**) Quantitative comparison of apoptosis between the static control and RPM groups. The results are expressed as means ± standard deviations. *p < 0.05, as compared with the static controls (*n* = 3).

To detect apoptotic cells, we used annexin V conjugated to fluorescein isothiocyanate (FITC) and flow cytometry. After 96 h, the percentage of total apoptotic cells was higher among the Jurkat/A4 cells in the RPM group (19.2% ± 4.2%) than in the static control group (10.1% ± 2.3%) (*n* = 3; *p* < 0.05). In contrast with the Jurkat/A4 cells, the percentage of total apoptotic cells was higher in the static control group (27.7% ± 5.2%) than in the RPM group (12.1% ± 2.3%) (*n* = 3; *p* < 0.05). Figure 2 shows the representative results of apoptosis analyzed by flow cytometry and the quantitative comparison results.

### 2.3. Simulated Microgravity Disturbed Cell Cycle of Jurkat/A4 Cells 

**Figure 3:**
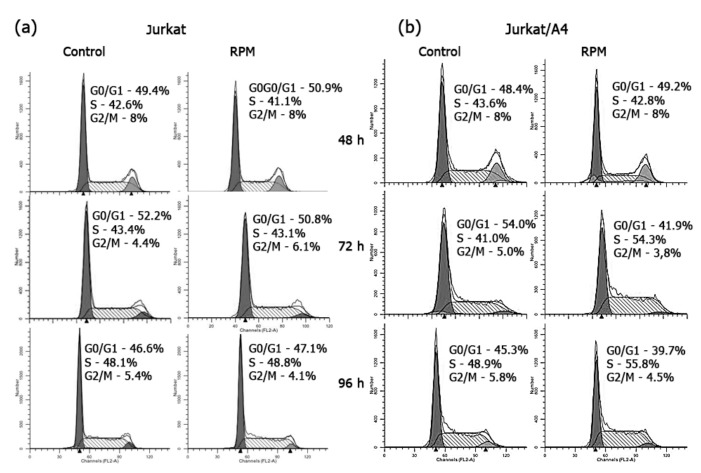

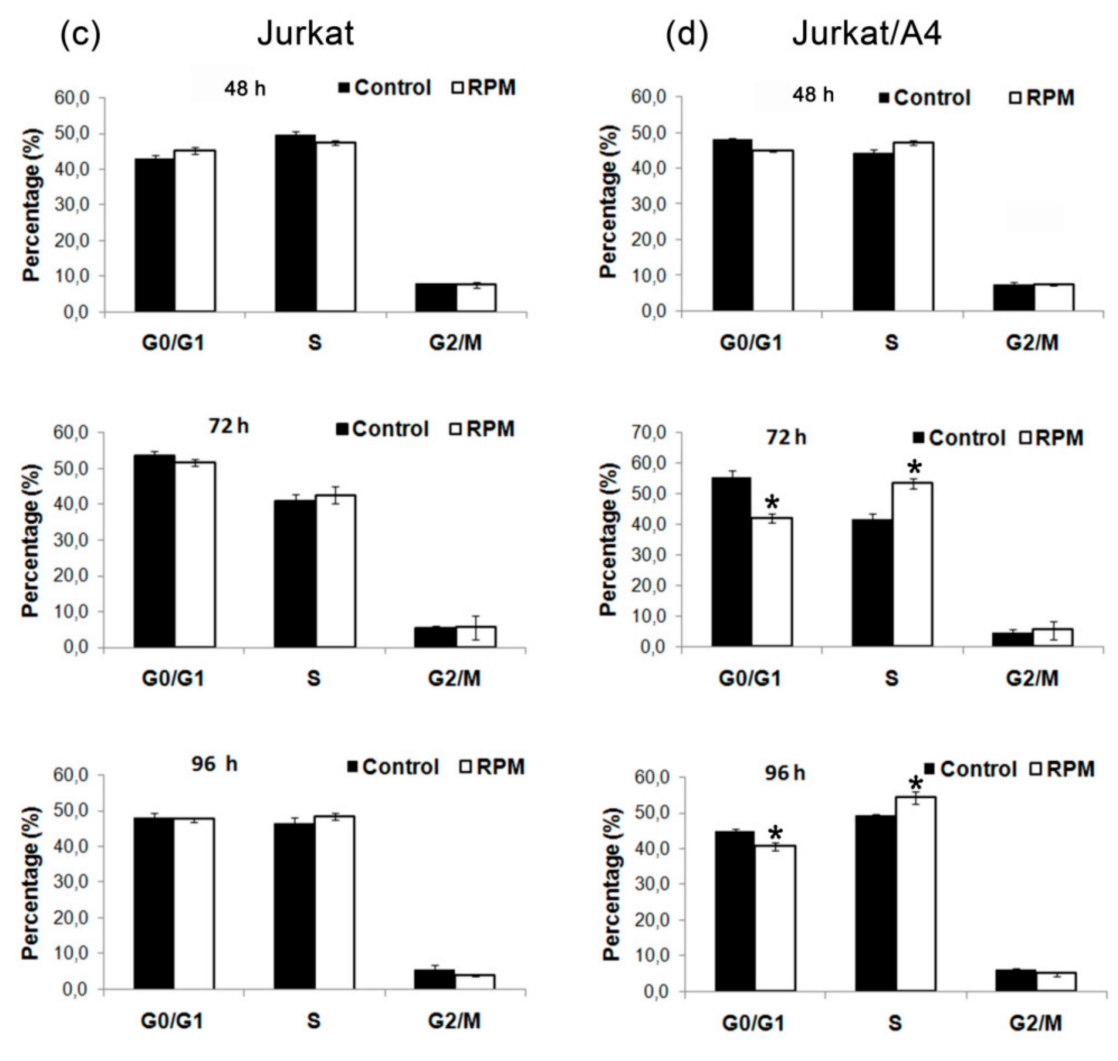
Effects of RPM-simulated microgravity on the cell cycle of Jurkat (**a**) and Jurkat/A4 cells (**b**). Cell cycle measurements were performed as described in the Materials and Methods section. Representative histograms are shown. Data are presented as a histogram, with cell number (*y*-axis) plotted against DNA content (*x*-axis). The first peak contains cells with diploid DNA in the G0/G1-phase. The cells in the second peak, with double PI-fluorescence intensity, were tetraploid in the G2-phase. The area between the two peaks represents cells in the S-phase. The number of cells gated in the G0/G1-, S-, or G2-phases is presented as a percentage of total cells (%). (**c**,**d**) Quantitative comparison of apoptosis between the static control and RPM groups of Jurkat and Jurkat/A4 cells. Percentages of populations in each cell cycle were averaged from five parallel experiments (at each time point). The results are expressed as means ± standard deviations. * *p* < 0.05, as compared with the static controls (*n* = 5).

Flow cytometry analysis showed that the percentages of Jurkat/A4 cells in the G0/G1-phase were 42.0% ± 1.6% in the RPM group and 55.3% ± 2.1% in the static control group, after 72 h of culturing (*n* = 5; *p* < 0.05). The number of Jurkat/A4 cells in the DNA synthesis-phase (S-phase) of the RPM group was significantly higher than that in the static control group (53.2% ± 1.6% vs. 41.3% ± 2.2%; *n* = 5; *p* < 0.05) (Figure 3). Additionally, the percentage of cells in the G0/G1-phase was 40.7% ± 1.1% in the RPM group in comparison with 45.1% ± 0.4 % in the static control group after 96 h (*n* = 5; *p* < 0.05). Further, the number of cells in the S-phase of the RPM group was higher than in the static control group after 96 h (54.3% ± 1.9% vs. 49.2% ± 0.3%; *n* = 5; *p* < 0.05). These results suggest that microgravity inhibited cell-cycle progression, arrested the cells at the S-phase of the cell cycle, and induced apoptosis in Jurkat/A4 cells. We observed no difference in the cell cycle between the experimental and control Jurkat cells.

### 2.4. Simulated Microgravity Changed the Expression of the Surface Marker ICAM-3 in Jurkat/A4 Cells 

**Figure 4:**
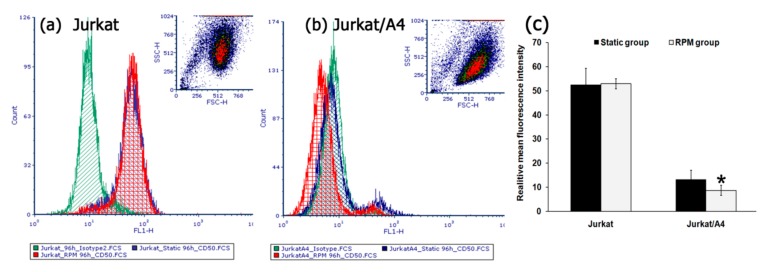
The effect of simulated microgravity on the expressions of intercellular adhesion molecule 3 (ICAM-3) also known as cluster of differentiation (CD)50 in Jurkat (**a**) and Jurkat/A4 cells (**b**) The expression of surface antigens was assessed by flow cytometry. The total number of cells expressing surface antigens on their cell surface was assessed by determining the (MFI) (**c**). Mouse IgG1-fluorescein isothiocyanate (FITC) was used as the isotype-matched control to exclude any nonspecific staining. The results are expressed as means ± standard deviations. * *p* < 0.05, as compared with the static controls (*n* = 5).

We examined the expression of the surface marker ICAM-3 for Jurkat cells vs. Jurkat/A4 cells. Normally, approximately 80% (Mean fluorescence intensity (MFI): 57.8) of the Jurkat cells showed expression of ICAM-3, as measured by flow cytometry. After 96 h of simulated microgravity there was no difference in the percentage of ICAM-3 on Jurkat cells between the RPM group and static control group. At the same time, the expression of the ICAM-3 surface marker on the RPM group Jurkat/A4 cells was lower (MFI: 7.2) than that among the static control cells (MFI: 13.7) (*n* = 5; *p* < 0.05) (Figure 4).

### 2.5. The Effect of Simulated Microgravity on the Morphology and Cytoskeleton of Jurkat/A4 Cells 

**Figure 5:**
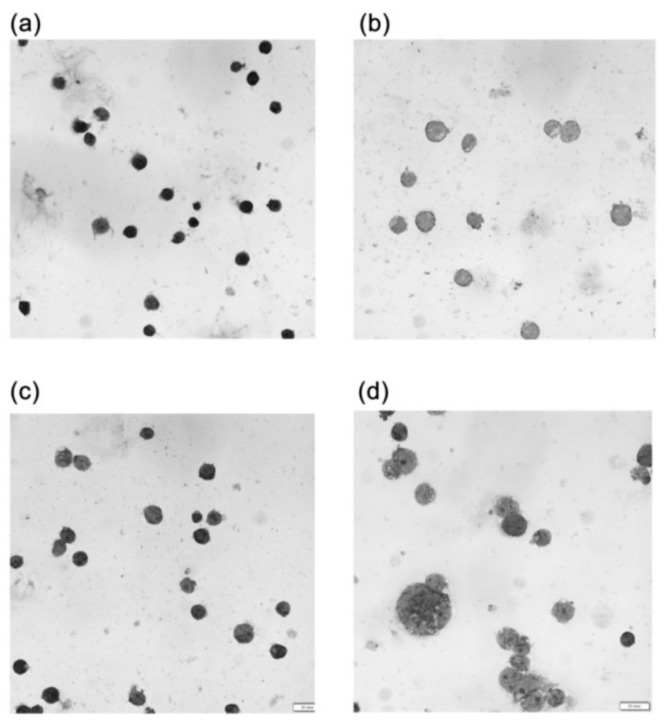
Morphological changes between Jurkat cells (**a**,**b**) and Jurkat/A4 cells (**c**,**d**). The smear results of cells stained with Giemsa solution can be seen. Morphological changes between the static control groups (**a**,**c**) and RPM groups (**b**,**d**) are apparent (96 h). As compared with the Jurkat static control cells, the size of the Jurkat/A4 cells increased after 96 h of RPM exposure. Photomicrographic images were obtained using an Olympus BX51 microscope equipped with an Olympus XM31 camera and software (Olympus, Tokyo, Japan). The original magnification was × 600. The scale bars indicate 100 µm.

Simulated microgravity had no significant effect on the morphology of either Jurkat or Jurkat/A4 cells after 24 or 72 h, demonstrating that cells grown under static culture conditions and under microgravity conditions show the typical morphology of Jurkat cells. However, after 96 h of RPM exposure, the size and shape of the majority of Jurkat/A4 cells had changed relative to the static control group cells (Figure 5).

**Figure 6:**
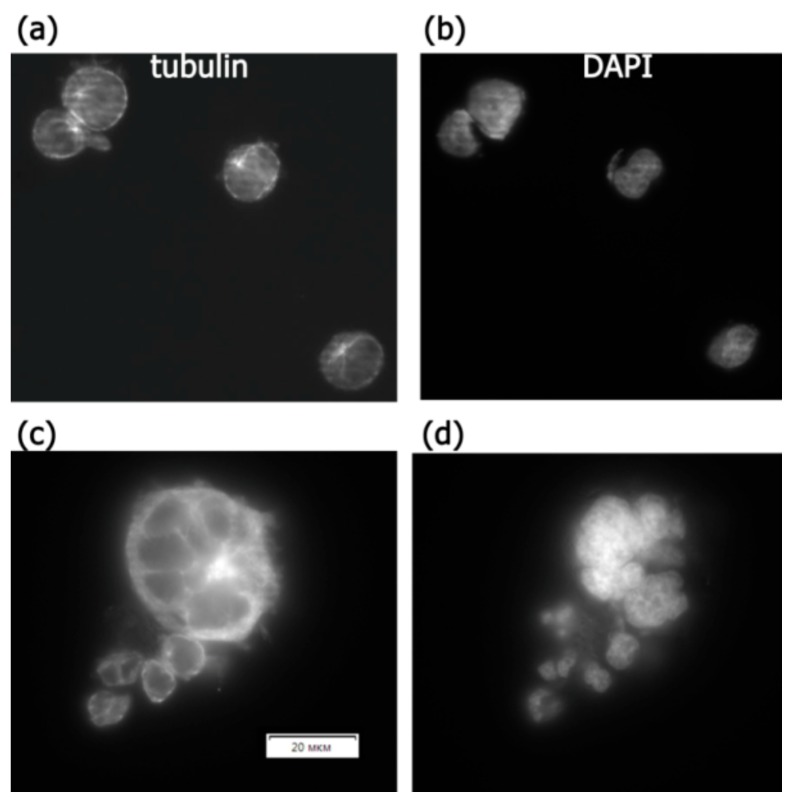
The effect of simulated microgravity on Jurkat/A4 cell microtubule cytoskeletons. The immunostaining of cells with beta-tubulin after paraformaldehyde fixation and saponin permeabilisation. (**a**,**b**) Static control group, 96 h; (**c**,**d**) RPM group, 96 h. Original magnification, ×400. The scale bars indicate 20 μm.

The immunocytochemical staining of tubulin-containing structures showed differences between the expression of beta-tubulin in the control group and the RPM group of Jurkat/A4 cells after 96 h. The expression level of beta-tubulin was reduced in Jurkat/A4 cells after 96 h of RPM exposure (Figure 6).

### 2.6. The Effect of Simulated Microgravity on Certain Apoptotic Proteins of Jurkat/A4 Cells 

**Figure 7:**
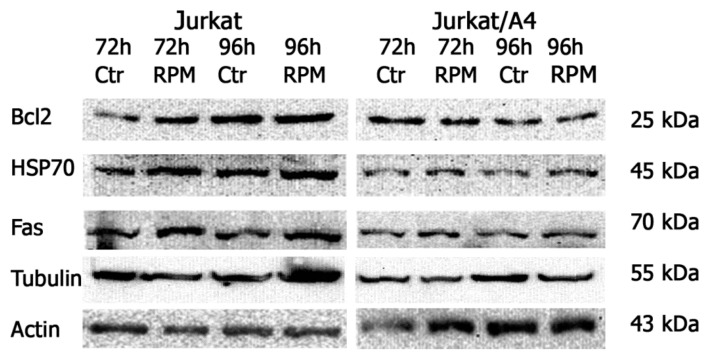
Jurkat cells and Jurkat/A4 cells, Western blotted with antibodies to anti-Bcl2, anti-HSP-70, anti- human Fas, and β-tubulin. The results are representative of three experiments.

To analyze apoptotic proteins, we examined the expression levels of Bcl-2, Fas, and HSP-70 in both Jurkat/A4 and Jurkat cells using Western blotting. Of note, the expressions levels of Bcl-2, Fas, and HSP-70 were higher in the Jurkat static control group than among the control Jurkat/A4 cells. However, the expressions levels of Bcl-2, Fas, and HSP-70 in these two kinds of cells under RPM-simulated microgravity conditions remained unchanged (Figure 7).

## 3. Discussion

Experiments performed under microgravity conditions have recently attracted increasing interest in many areas of biomedicine, such as oncology, cardiology, immunology, pharmacology, and regenerative medicine.

Most studies indicated that cells of the immune system are highly sensitive to altered gravity [16,17,18]. Jurkat cells are stable cellular models easily able to be managed, and lymphocytes can be considered as biosensors of the whole-body response and of adaptation to environmental conditions [16,28]. As reviewed by Morabito et al., shear stress during rotation, and the use of a depleted medium that cannot be renewed, impacts the outcomes of exposure lasting longer than 96 h [16]. According to the results of the gene expression profile, after the first 24 h of exposure to stimulated microgravity, proliferating Jurkat cells adapted to the new environmental condition, showing a healthy status [29].

In this study, we investigated the effects of simulated microgravity on Jurkat cells and their multidrug-resistant subline Jurkat/A4 cells. Our experiments indicated that the percentage of trypan blue-stained dead Jurkat/A4 cells increased under RPM-simulated microgravity conditions, relative to static Jurkat/A4 cells. Confirmation of dead Jurkat/A4 cells was achieved by viewing the flow cytometry and annexin V, which showed that after 96 h, the percentage of total apoptotic cells was higher among the RPM-treated Jurkat/A4 cells than in the static Jurkat/A4 group. 

Some researchers have reported that microgravity exposure changes the morphological phenotype, cell cycle, and cell adhesion, and has an impact on cell survival that is highly dependent upon the cell type, as well as on the duration of exposure [30,31]. Long-term RPM exposure of human endothelial cells induced the formation of three-dimensional tubular structures and spheroids [32]. Previous reports have shown that simulated microgravity induced partial arrest in the G2/M-phase of the MCF-7 cells and normal murine vascular smooth muscle cells, and also slowed down the cell cycle progression of myoblasts [33]. 

However, exposure of malignant glioma cells to simulated microgravity for 3 days did not cause cell-cycle arrest or a change between the control and clinostat groups [34]. In our earlier study, we reported that simulated microgravity inhibited proliferation, induced apoptosis, and changed the morphology of MEG-01 megakaryoblastic cells [35]. In the present study, we demonstrated that the percentage of Jurkat/A4 cells in the G0/G1-phase decreased after 72 and 96 h. At the same time, the percentage of cells in the S-phase increased in the RPM group in comparison with in the static control group. These data indicate that microgravity inhibits the cell-cycle progression of human multidrug-resistant Jurkat/A4 cells.

ICAMs are transmembrane proteins that are expressed on epithelial cells, endothelial cells, and cells of the immune system, including T-cells and macrophages. ICAM-1 can be considered as a rapid-reacting and sustained gravity-regulated molecule in mammalian cells [36,37]. TNBC (triple-negative breast cancer) cells exposed to short-term microgravity obtained by parabolic flight maneuvers kept all signs of a more aggressive phenotype, as elevations of ICAM1 and Vascular cell adhesion protein 1 (VCAM1) proteins occurred quickly. 

ICAM-1 expression was heightened in macrophage-like differentiated human U937 cells during the microgravity phase of parabolic flights and in long-term microgravity provided by a two-dimensional clinostat or during the orbital SIMBOX/Shenzhou-8 mission [32,36]. Disturbed immune function in microgravity could be a consequence of ICAM-1 modulation in the monocyte/macrophage system, which, in turn, could have a strong impact on the cells’ interaction with T-lymphocytes and migration [36].

Short-term microgravity led to an increased synthesis of ICAM-1 and VCAM-1 proteins in MDA-MB-231 breast cancer cells during a parabolic flight campaign [31]. Modulation of the expression of surface adhesion molecules such as ICAM-1 has been reported as a consequence of long-term microgravity of cultured human endothelial cells [38,39]. 

Intercellular adhesion molecule 3 (ICAM-3, also known as CD50), a human leukocyte-restricted immunoglobulin super-family (IgSF) member, has previously been implicated in apoptotic cell clearance. ICAM-3 can function as a phagocytic marker of apoptotic leukocytes, on which it acquires altered macrophage receptor-binding activity. ICAM-3 also inhibits lymphocyte survival through the induction of apoptosis and promotion of adhesion of apoptotic leukocytes to phagocytic macrophages [40].

An apoptosis-associated reduction in ICAM-3 results from the release of ICAM-3 within microparticles that potently attract macrophages to apoptotic cells. Taken together, these data suggest that apoptotic cell-derived microparticles, bearing ICAM-3, promote macrophage chemoattraction to sites of leukocyte cell death, and that ICAM-3 mediates subsequent cell corpse tethering to macrophages. The defined function of ICAM-3 in these processes, and profound defect in chemotaxis noted to ICAM-3-deficient microparticles, suggest that ICAM-3 may be an important adhesion molecule involved in chemotaxis to apoptotic human leukocytes [41]. 

Though our results showed a slight decrease of the expression of the ICAM-3 surface marker on the RPM group in Jurkat/A4 cells, in comparison to the control, we conclude that ICAM-3 also may be an important adhesion molecule involved in the induction of leukocyte apoptosis and the functions of cell growth behavior in microgravity conditions. As demonstrated recently, the transitory effects induced by stimulated microgravity on cell shape and proliferation are accompanied by metabolic changes that are, above all, related to mitochondrial activity [16]. The modification of external forces caused by stimulated microgravity conditions induced transient modifications in cell shape and intracellular density in Jurkat cells, accompanied by transient changes in the expression levels of some cytoskeletal proteins such as actin, tubulin, and vimentin, which are not only structural proteins but also the main functional milestones in cytoskeleton dynamics [42,43].

**Figure 8:**
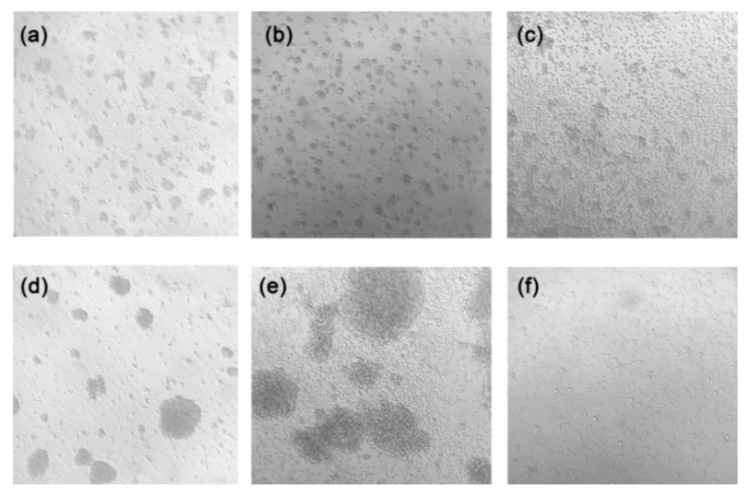
Bright-field images of Jurkat cells and Jurkat/A4 cells using the Carl Zeiss Axiovert 40 CFL microscope (Carl Zeiss AG, Oberkochen, Germany) at ×100 magnification. Jurkat cells are shown (**a**) before seeding in the flask, (**b**) as static controls (96 h), and (**c**) in the RPM group (96 h). Jurkat/A4 cells are shown (**d**) before seeding in the flask, (**e**) as static controls (96 h), and (**f**) in the RPM group (96 h).

Notably, that morphological differences between Jurkat cells and Jurkat/A4 cells were observed after 96 h of microgravity exposure, whereas the size of Jurkat/A4 cells increased and their cellular shape was changed in the RPM group. Interestingly, as seen in vials of Jurkat/A4 cells after microgravity exposure, the cells were no longer grouped (Figure 8). Additionally, the expression level of beta-tubulin was reduced in Jurkat/A4 cells after 96 h of RPM exposure, as shown by immunofluorescence analysis. These experiments support the conclusion that simulated microgravity has different effects depending upon the cell type. 

There are numerous studies regarding the effects of microgravity on T-cells, encompassing a large variability in cell models and environmental conditions. Many variations in the experimental plan also exist even when selecting the studies covering only Jurkat cells (e.g., different media composition, exposure to real or simulated microgravity, and time of exposure) [15]. Apoptotic signaling is enhanced in cancer cells subjected to microgravity. Recently, it was demonstrated that simulated microgravity altered the cytoskeleton and nuclear positioning, leading to enhanced cell apoptosis via suppressing the focal adhesion kinase (FAK)/RhoA-controlled mammalian target of rapamycin complex 1 (mTORC1)/nuclear factor-kappa B (NF-κB) and extracellular signal-regulated kinase1 (ERK1/2) pathways [44]. Elsewhere, microgravity altered cis–diamminedichloride platinum (II) (CDDP) sensitivity through the activation of caspase-3 by a p53-independent mechanism in the hepatoblastoma cell line, HepG2 [20]. Microgravity decreased the levels of mTOR and increased the microtubule-associated protein light chain 3-II/I ratio (LC3), suggesting the activation of autophagy. Disturbed immune function in microgravity could be a consequence of ICAM-1 modulation in the monocyte/macrophage system, which, in turn, could have a strong impact on the cells’ interaction with T-lymphocytes and migration [20].

Simulated microgravity can also induce the nuclear translocation of Bax and Bcl-2 in glial cultured C6 cells [45]. Simulated microgravity altered the cytoskeletons and promoted cell apoptosis through suppressing the NF-κB pathway, leading to up- and down-regulated pro-apoptosis (caspases 3, 7, and 8) and anti-apoptosis (Bcl-2 and Bnip3) molecules, respectively [44].

We did not observe a change in the expression levels of Bcl-2, Fas, HSP-70, or beta-tubulin in either Jurkat or Jurkat/A4 cells under RPM-simulated microgravity conditions. However, the expressions of Bcl-2, Fas, and HSP-70 were higher in the Jurkat static control group than among the control Jurkat/A4 cells, corresponding with the findings of our previous studies [26,27]. 

Therefore, our data demonstrated for the first time that the Jurkat/A4 cells with an acquired multidrug resistance phenotype are more sensitive to RPM microgravity, possibly due to the fact that Jurkat cells are a cancer p53-deficient cell line [46]. In our previous work, the results suggested that CD73 is one component of a complex nonspecific antiapoptotic program, which was, in our case, upregulated during selection for Fas resistance [27]. This could be important in order to understand the role of the expression factors associated with NF-κB and MAPK signaling pathways. We can also speculate about the precise mechanism; perhaps Jurkat/A4 has its own mechanism of death similar to anoikis [47]. 

Scientists search for new ways to identify targets for novel drugs, and some of them have considered using experimentation in altered gravity conditions [19,48,49,50]. The microgravity environment might also be useful for enhancing the development of complex tissues composed of multiple cell types [51]. The unique combination of a 3D microfluidic tissue chip and a microgravity environment can provide unique insights into disease models and therapeutic targets. For example, the development of a biomimetic kidney organoid has been the focus of intense investigation [52]. 

In summary, we revealed morphological changes, changes in the surface expression of intercellular adhesion molecule 3, changes in the induction of apoptosis, and changes in the inhibition of cell-cycle progression, for human multidrug-resistant Jurkat/A4 cells exposed to a random positioning machine. 

## 4. Materials and Methods 

### 4.1. Cell Culture

The Jurkat cell line (clone EG-1) was obtained from the American Type Culture Collection (Manassas, VA, USA). A multiresistant cell line (Jurkat/A4) was prepared from this Jurkat cell line by serial treatment with apoptosis-inducing anti-CD95 monoclonal antibody [26]. The cells were maintained at 37 °C in an atmosphere of 95% air and 5% CO_2_ as suspension cultures in RPMI 1640 medium supplemented with 10% fetal bovine serum (FBS) and 5 μg/mL of gentamicin (all reagents from Life Technologies, USA). 

### 4.2. RPM

Microgravity conditions were simulated using an RPM (Dutch Space; Astrium EADS, Leiden, the Netherlands) [14]. To start an RPM experiment, Jurkat and Jurkat/A4 cells with a density of 2 × 10^6^ cells/mL were seeded in T12.5 flasks (Jet Biofil) completely filled with culture medium to avoid air bubbles and to minimize liquid flow, and were cultured at 37 °C in a humidified atmosphere of 95% air and 5% CO_2_. The culture flasks were fixed onto the desktop RPM at the center of the platform, which was rotated at a speed of 60°/s. The static culture flasks (1 g control) were placed in the same culture incubator. 

### 4.3. Cell Viability Analysis

Cell viability was assessed by vital dye exclusion (trypan blue; Invitrogen, Carlsbad, CA, USA). After incubation for indicated times (24, 48, 72, and 96 h), cells were removed from the culture flasks and an aliquot of the suspension (20 μL) from each sample was mixed with an equal amount of 0.4% (*w*/*v*) trypan blue dye and placed on the surface of the working slide for analysis on an automated cell counter (Countess; Invitrogen, Carlsbad, CA, USA). Each test sample of the cells was counted in duplicate.

### 4.4. Cell-Cycle Analysis

The cell-cycle distribution of cells was analyzed by Propidium-iodide (PI) staining of the cellular DNA content and the percentages of cell population in the G0/G1-, S-, or G2/M-phases were calculated from histograms created by the CellQuest software (BD Biosciences, Franklin Lakes, NJ, USA). After incubation for the indicated times (48, 72, and 96 h), cells were removed from the culture flasks, washed in phosphate-buffered saline (PBS), gently fixed in ice-cold 70% ethanol, and kept for 24 h at 4 °C. 

The detection method was based on the use of a commercial kit (BD Biosciences, Franklin Lakes, NJ, USA). Briefly, following fixation, the cells were washed twice with PBS and resuspended in the staining buffer containing PI and RNase. The samples were incubated for 30 min in a dark place at room temperature (RT). Cell-cycle analysis was performed with the flow cytometer FACSCalibur (Becton Dickinson, Franklin Lakes, NJ, USA) with an air-cooled argon laser (wavelength: 488 nm). A minimum of 25,000 events were analyzed for each sample. Each test (time point) sample of cells was counted in duplicate. Flow cytometric data files were collected and analyzed using the CellQuest software. Histograms generated by FACSCalibur were analyzed using the ModFit Cell Cycle Analysis Software V2.0 (Verity, Topsham, ME, USA) to determine the percentage of cells in each phase (G0/G1, S, and G2). 

### 4.5. Apoptosis Analysis

Cell apoptosis analysis was performed using an annexin V-FITC apoptosis detection kit I (BD Biosciences, Franklin Lakes, NJ, USA) according to the manufacturer’s instructions. Briefly, 1 × 10^6^ cells were washed once with cold PBS, suspended in 100 µL of binding buffer containing FITC-conjugated annexin V and PI, and incubated for 15 min on ice and in the dark, per sample. The samples were analyzed by flow cytometry with FACSCalibur. A minimum of 15,000 events were analyzed for each experimental sample.

### 4.6. Flow Cytometry Immunophenotyping

The expression of ICAM-3 was analyzed using fluorochrome-coupled monoclonal human antibody: FITC-coupled anti-CD50-FITC (BD Biosciences, Franklin Lakes, NJ, USA). A mouse immunoglobulin G isotype-matched control conjugated to FITC was used to exclude nonspecific binding. Antibodies were prediluted for use at the recommended volume per test. 

The cells were removed from the flasks, twice washed in PBS, and incubated for 30 min at RT in the dark with monoclonal antibodies and isotype-matched controls. In each test, monoclonal antibodies were added to 0.5 to 1 × 10^6^ cells in a 100 µL experimental sample. All samples were analyzed using flow cytometry. At least 10,000 events were acquired for each experimental sample. The positive analysis region was determined using the negative isotype control. The MFI and the percentage of cells expressing CD50 were then determined.

### 4.7. Observation of Morphological Changes 

The cells were collected on different days of culture and washed with PBS, fixed with 100% methanol, and then stained with Giemsa solution for five minutes. Morphological changes in the samples were analyzed using an Olympus microscope BX51 equipped with an Olympus DP71 camera, 60/1.35 oil Apolens, and the cellSens version 2.1 image analysis program (all Olympus, Tokyo, Japan).

### 4.8. Western Blotting

Cells were collected, washed with PBS twice, and were lysed in radioimmunoprecipitation assay buffer (50 mM of Tris, 150 mM of NaCl, 0.5% sodium deoxycholic acid, 1% (v/v) Nonyl Phenoxypolyethoxylethanol-40 (NP-40), 0.1% (w/v) sodium-dodecyl sulfate (SDS); pH: 7.4). Protein concentrations were determined using the Bradford method, using a NanoDrop 1000 spectrophotometer (Thermo Fisher Scientific, Waltham, MA, USA). Next, 10 to 20 µg of total protein per sample was used for 12% to 15% sodium-dodecyl sulfate–polyacrylamide gel electrophoresis and semidry transfer to a 0.45 µm polyvinylidene difluoride membrane (Immobilon-P Transfer Membran; Millipore, Burlington, MA, USA). Membranes were blocked by 5% milk (blotting-grade blocker; Bio-Rad Laboratories, Hercules, CA, USA) in TBST buffer at RT for 1 h and then treated overnight at 4 °C with working dilutions of respective primary antibodies in the presence of 5% milk with 0.06% NaN_3_ in TBST buffer. 

Membranes were hybridized with a mouse monoclonal antihuman Bcl-2 (1:500 dilution; BD Biosciences, Franklin Lakes, NJ, USA), mouse monoclonal anti–beta-tubulin (1:1000 dilution; Abcam, Cambridge, UK), mouse monoclonal anti-Hsp-70 (1:5000 dilution; Abcam, Cambridge, UK), mouse monoclonal anti-human Fas (1:1000 dilution; MBL, Tokyo, Japan), mouse monoclonal anti–beta-tubulin (1:1000 dilution, Abcam, Cambridge, UK), and mouse monoclonal anti-actin Ab-5 (1:10,000 dilution; BD Biosciences, Franklin Lakes, NJ, USA) separately. 

Following incubation with primary antibodies, the membranes were washed with TBST for 10 min, four times, and were then incubated with secondary horseradish peroxidase-conjugated goat antimouse immunoglobulin G (1:2000 dilution; Thermo Fisher Scientific, Waltham, MA, USA) at RT for one hour with subsequent washing in TBST for 10 min, four times. The detection of bands was implemented using the Kodak 440CF image station and Amersham ECL Western blotting detection kit (GE Healthcare, Chicago, IL, USA) according to the manufacturer’s instructions. A mouse monoclonal anti-actin Ab-5 antibody (1:1000 dilution; BD Biosciences, Frankin Lakes, NJ, USA) was used as a loading control.

### 4.9. Immunocytochemistry

For immunostaining, the cells were washed in PBS, fixed in 4% paraformaldehyde for 10 min, and permeabilized with 0.5% saponin for 15 min according to the antibody protocol. After washing with PBS, the cells were incubated in blocking buffer (10% FBS) for two hours. Following a further wash with PBS, the cells were incubated with anti-beta-tubulin (1:1000 dilution; Abcam, Cambridge, United Kingdom) overnight at 4 °C. The cells were washed three times with PBS and incubated for 15 min with 4′,6-diamidino-2-phenylindole (DAPI) at RT. Then, the cells were washed three times with PBS and mounted on glass slides using a mounting medium (Thermo Fisher, Scientific, Denmark). Fluorescence was visualized using a BX51 microscope equipped with an Olympus DP71 camera, 60/1.35 oil Apolens, and the cellSens version 2.1 image analysis program (all Olympus, Tokyo, Japan).

### 4.10. Statistical Analyses

Data are presented as means ± standard deviations of at least three independent experiments. Differences between groups were assessed using Student’s *t*-test and the Mann–Whitney U test. Values of *p* < 0.05 were considered as statistically significant. 

## 5. Conclusions

With this research, we conclude that Jurkat/A4 cells are more sensitive to RPM-simulated microgravity in comparison with the parental Jurkat cell line. We suggest that intercellular adhesion molecule 3 also may be an important adhesion molecule involved in the induction of leukocyte apoptosis. In addition, our results are consistent with other studies, in that the using different cell cultures under simulated microgravity will help both for the discovery of molecular mechanisms of cancer and for in vitro therapeutic screening [48,49,50,51,52]. 

It is, therefore, the case that Jurkat/A4 cells with an acquired multidrug resistance phenotype could be a useful model for studying the effects of simulated microgravity and testing anticancer drugs. Future studies on the mechanisms and physiological implications of this phenomenon are currently under construction.

## Abbreviations

3D	Three-dimensional
Abs	Antibodies
BCL2	B-cell lymphoma 2
BNIP3	BCL2 interacting protein 3
CD50	ICAM-3 also known as cluster of differentiation (CD)50
CD95	Apo-1/Fas (CD95), a receptor of the TNF Receptor (TNFR) family
CDDP	*cis*-diamminedichloridoplatinum(II)
Con-A	Concavalin A
DAPI	4′,6-diamidino-2-phenylindole
ERK1	Extracellular signal-regulated kinase1
FAK	Focal adhesion kinase
FAS	FS-7-associated surface antigen, also known as apoptosis antigen 1 (APO-1)
FBS	Fetal bovine serum
FITC	Fluorescein isothiocyanate
ICAM-3	Inter-cellular adhesion molecule 3
LC3	Microtubule-associated protein light chain 3-II/I ratio
MAPK	Mitogen-activated protein kinase
MEG-01	megakaryoblastic cells
MFI	Mean fluorescence intensity
MCS	Multicellular spheroid
mTORC1	Mammalian target of rapamycin Complex 1
NF-κB	Nuclear factor-kappa B
NP-40	Nonyl Phenoxypolyethoxylethanol-40
PBS	Phosphate-buffered saline
PI	Propidium-iodide
RhoA	Ras homolog family member A
RPM	Random positioning machine
RT	Room temperature
SDS	Sodium-dodecyl sulfate
SDS-PAGE	SDS -polyacrylamide gel electrophoresis
TBST	Tris-buffered saline buffer with Tween 20
VCAM1	Vascular cell adhesion protein 1
